# Economic Analysis of Patient’s Own Medication, Unit-Use and Ward Stock Utilization: Results of the First Pilot Study

**DOI:** 10.3390/ijerph191811350

**Published:** 2022-09-09

**Authors:** Hamimatul Hayat Abdul Nasir, Hui Poh Goh, Daniel Vui Teck Wee, Khang Wen Goh, Kah Seng Lee, Andi Hermansyah, Yaser Mohammed Al-Worafi, Long Chiau Ming

**Affiliations:** 1 PAPRSB Institute of Health Sciences, Universiti Brunei Darussalam, Gadong BE1410, Brunei; 2 Pharmacy Department, Suri Seri Begawan Hospital, Ministry of Health, Belait, Kuala Belait KA1131, Brunei; 3 Faculty of Data Sciences and Information Technology, INTI International University, Nilai 78100, Malaysia; 4 Faculty of Pharmacy, University of Cyberjaya, Cyberjaya 63000, Malaysia; 5 Department of Pharmacy Practice, Faculty of Pharmacy, Universitas Airlangga, Surabaya 60115, Indonesia; 6 College of Medical Sciences, Azal University for Human Development, Amran P.O. Box 447, Yemen; 7 College of Pharmacy, University of Science and Technology of Fujairah, Fujairah P.O. Box 2202, United Arab Emirates

**Keywords:** pharmacy, medicine management, sustainability, wastage, medication safety

## Abstract

Background: Medication wastage is causing a cost burden to the healthcare system that is worth millions of dollars. An economic and ecological friendly intervention such as using a patient’s own medications (POM) has proven to reduce wastage and save the cost spent by the hospital. The potential benefits of using POM in inpatient settings have yet to be explored in a country with universal health coverage. This study aimed to pilot test the POM intervention in an adult ward setting and to perform the economic analysis of using POM and ward stock during hospitalization. Methods: A prospective cross-sectional observational study was conducted among the patients admitted to the medical and surgical wards in a public hospital located in Brunei Darussalam between February 2022 and April 2022. Hospitalized adults above 18 years old with regular medications with a minimum length of stay of 48 h and a maximum length of stay of 21 days were included in the study. These eligible patients were divided into a POM group and a non-POM group. The economic analysis of using POM was performed by calculating the direct cost per unit of medication used during admission (from unit-use, ward stock and POM) and comparing the cost spent for both groups. Expired ward stock deemed as medication wastage was determined. Medical research ethics were approved, and all participating patients had given their written informed consent before enrolling in this study. Results: A total of 112 patients aged 63.2 ± 15.8 years participated in this study. The average cost of medication supplied by the inpatient pharmacy for the non-POM group was USD 21.60 ± 34.20 per patient, whereas, for the POM group, it was approximately USD 13.00 ± 18.30 per patient, with a mean difference of USD 8.60 ± 5.17 per patient (95% CI: −3.95, 27.47, *p* ≥ 0.05). The use of POM minimized 54.03% (USD 625.04) of the total cost spent by the hospital for the POM group within the period of the study. Conclusion: The pilot study showed that the supplied medication cost per patient was not significantly different between the POM and non-POM groups. Nevertheless, the utilization of POM during hospitalization is capable of reducing at least 50% of the total cost spent on inpatient medications by the hospital. The use of POM during hospitalization also helped in reducing the total time spent on the medication process per patient.

## 1. Introduction

Medication wastage has been an issue that has been increasing over the years and is widely reported in the literature, exposing the need to address this issue [1,2]. It was accentuated by the World Health Organization (WHO) that fifty percent of the medications supplied were neglected and wasted [3]. As a result, medication worth approximately GBP 300 million in the United Kingdom and USD 2.4 billion in the United States are wasted per year [3].

A patient’s own medication (POM) can be described as the medications brought during hospitalization, which patients acquired from the hospital’s prescription or in community settings [4]. The introduction of POM has shown positive impacts in terms of cost, reducing adverse events and improving treatment outcomes [5]. As highlighted, the use of POM in hospitals has shown positive yields, especially in terms of cost-saving. A previous study in eleven wards of a public hospital located in Southampton, England, showed a significant annual cost saving of GBP 24,213 after the implementation of POM during hospitalization [6]. Another similar study conducted at a private hospital located in Western Australia estimated a total of 9.9% of annual savings from the utilization of POM in inpatient settings [7]. On the other hand, in Malaysia, the implementation of POM has shown a significant reduction in total cost spent by the hospital, by USD 114.27 per patient per year [3]. According to the report from a Canadian Hospital, the use of POM for 3 weeks has extrapolated a total cost saving of CAD 1600 [8]. Although, in fact, the estimated cost saved by the hospital with the use of POM is not on par with newly supplied medications, this intervention is still significant and worth being further investigated in helping to reduce medication wastage and cost [9]. A study in a specialist children’s hospital located in London, England, reported that three-quarters of the patients that brought POM during hospitalization were discharged with at least one of the same medications before they were hospitalized, while the rest were discharged with the same medications prior to hospitalization [10]. The implementation of POM within the two weeks of the study saved the hospital a total of GBP 5549 [10]. Another study in a hospital in Canada also mentioned that 90% of POMs were more likely to be used for their inpatient care [8]. Therefore, since the use of patients’ regular medications during admission is quite significant, the implementation of POM is feasible. However, some patients lack awareness in bringing their own medications during admission, as found in a previous study where only half of the warded patients brought their own medications, and 25% of them only brought their POM after a reminder [4]. Some patients admitted that they did not bring their medications because they were not informed to bring their own regular medications [4]. Hence, the encouragement and awareness of POM use in inpatient settings should be raised among healthcare professionals, patients and caregivers [10].

Medication dispensing systems vary, yet they all have the ultimate goals of ensuring patients receive the best possible treatment. In the current study, a unit-use supply system was used that could lead to medication wastage if the earlier prescribed drugs are not finished due to a change in prescriptions or not being returned to the pharmacy. On the other hand, a unit-dose supply system was recommended to reduce potential medication errors, albeit at higher operational and labor requirements [11]. Investigations of these different dispensing systems were performed to find the errors made when compared using the unit-dose system by the pharmacist versus the ward stock system by the staff nurse [12]. Often the ward stock (also known as floor stock) included the leftover or unused medications that stay idle and expire. This tends to lead to medication wastage because of the potential change in prescription due to patient disease progression and continuous monitoring [11].

Although the use of POM during hospitalization requires thorough quality checking to ensure that the medications are still within standard and safe to be used, this intervention has been approved and applied by 90% of the pharmacy directors from 300 small hospitals that were registered under the American Hospital Association [13]. These studies have demonstrated the benefits and feasibility of the use of POM in the wards. Considering that this intervention has not been well studied in the Asian region, including using an observational study design, we conducted a pilot study to investigate the financial impact of using POM and ward stock in inpatient settings. This study also helped to describe the cost involved in the use of ward stock.

## 2. Methods

A cross-sectional observational study was conducted in an inpatient setting at one public hospital located in the district of Belait in Brunei Darussalam from February 2022 to April 2022. There are, in total, three wards of the hospital, of which two surgical wards and one medical ward participated in this study. This study was approved by the PAPRSB Institute of Health Sciences Research Ethics Committee (Reference Number: UBD/PAPRSBIHSREC/2021/76) prior to the conduction of the study. In addition, this study was performed according to the Principles of the Declaration of Helsinki with proper documentation of patient consent and protocol. 

### 2.1. Study Participants

Eligible participants were adults above 18 years old with regular medications admitted to the medical ward (merged with a zoning of male and female), female surgical ward and male surgical ward between February 2022 and April 2022, with a minimum length of stay of 48 h and a maximum length of stay of 21 days. Hence, patients with no regular medications, on repetitive admission and a longer admission period of more than 21 days were excluded from this study. The term ‘regular medications’ here refers to medications that the patient takes on a daily basis, such as for chronic illnesses where repeat prescriptions are given. Some common examples of chronic diseases are diabetes mellitus, dyspepsia or hypertension.

### 2.2. Study Design

The eligible patients’ medication records were screened via the electronic hospital information system (e-HIS) and the physical ward medication administration record by the researchers post identification of their POM status in the ward. For the POM group, the POM would be handed to the nurses in charge. The POM was kept in the respectively labeled compartment inside the medication trolley. The POM group, on top of their medication brought from home, were prescribed other medications too, depending on their treatment needs. 

Throughout the study period, the researcher checked through the ward medication list to determine which was POM, from inpatient pharmacy supply or from ward stock. Ward stock here refers to the floor stock kept in the wards. For the non-POM group, the list of medication supplied by the inpatient pharmacy and the ward stock used were identified. Dispensation was carried out daily in all medical wards, except on Sunday, when drugs were dispensed for a three-day supply. There are some exceptions to the unit-dose drug dispensing system: the distribution of large volume solutions, antiseptics and disinfectants. The prescription of these drugs was not included in our study.

At the selected ward, nurses were responsible for the drug inventory within their unit. Restocking the ward stock was performed via approved lists and was supplied by the inpatient pharmacy on an ad hoc basis. This system normally co-exists with the ward stock distribution system, for which the respective ward had its own in-house ward stock medication list. The doctors wrote prescriptions using a computerized physician order entry system as part of the e-HIS. The nurses were tasked to give the ordered medication to the patient in the ward based on the bed head ticket. In the unit of use system, for example, drug A tablet once daily was prescribed, and the patient requires one tablet each day. Therefore, by using a unit of use on three days basis, three tablets were supplied as unit-use on day one. On day three, if drug A was still continued, the nurses would request a drug top-up. Nurses would collect the prescribed medicine and store it in the medication trolley prior to administering the medicine to the respective patient. 

The eligible warded patients were categorized into POM and non-POM groups. The non-POM group consisted of patients that were on regular medications but did not bring their medication during their admission, whether intentionally or not. Whereas the POM group were those patients that brought their own medication during admission, on their own or after being reminded by the nurses. Thus, any regular medication that was used during admission for patients in the non-POM group was provided by the inpatient pharmacy or from the wards via the ward stock. On the other hand, the POM group used their own medication throughout their stay at the hospital. Since ward stock was used during patient admission, the usage of ward stock was also monitored in this study.

### 2.3. Cost Analysis of Medication (POM, Unit-Use, Ward Supply)

Cost analysis was carried out in order to find the cost impact of POM use during hospitalization. The medication cost per unit was standardized based on the latest medicine price list fixated by the Department of Pharmaceutical Services, Brunei Darussalam, for the year 2021 in Brunei Dollar (BND). The cost of drugs was converted to USD based on BND 1.00 equal to USD 0.74 for the year 2022.

The cost impact of using POM was then determined by determining the cost per unit of medication used during admission and comparing the cost spent for both groups. To determine the cost of medication used, the patient’s list of medications and medication administration chart via electronic medical record, Hospital Information System (HIS) to monitor the medications’ name, strength and quantity used by the patient during admission were recorded on a daily basis. On another note, the medications taken into account in this study were limited to prescribed medication only; medication such as injections and over-the-counter (OTC) medicines that can be used within the inpatient settings or at home were not included.

For the non-POM group, the cost of medication use per patient was calculated based on the quantity of medicines supplied by the inpatient pharmacy, while for the POM group, the quantity of both patient’s own medication and any medicines supplied by the inpatient pharmacy used during admission were both determined. Since the study from van Herpen-Meeuwissen et al. [14], was used as a reference, the cost of medications used for both groups was calculated using adjusted patient days for comparison as used in the referenced study. Individual detailed medication use information, such as a list of medications, timing and dosage of the medications, were displayed under the medication administration chart section in e-HIS. 

Equation (1) was used to calculate the cost of medication used for both groups per adjusted patient days [15].

(1) $$\;\frac{Medication\;price\;per\;unit\;used\;}{Average\;length\;of\;stay}\times 100\;days$$

Equation (2) was used to calculate the real cost saving for using POM:

(2) $$\frac{\begin{array}{c} Total\;cost\;of\;the\;regular\;medications\;prescribed\;during\;hospitalization\;\\ -\\ Cost\;of\;the\;medications\;used\;by\;the\;patient\;during\;hospitalization\;\; \end{array}}{Average\;length\;of\;stay}\times 100\;days$$

Equation (3) was used to calculate the expected cost saved if POM were used:

(3) $$\frac{Cost\;of\;the\;regular\;medications\;supplied\;\;during\;hospitalization}{Average\;length\;of\;stay}\times 100\;days$$

Meanwhile, the cost of wastage refers to the total cost of medications that were unused and left in the trolley expired.

### 2.4. Statistical Analysis

All data were entered into Microsoft Excel to summarize the descriptive statistics, which were then imported to RStudio for analysis. The normality of the datasets was tested using Shapiro–Wilk test, where *p* ≥ 0.05 indicates that the data were normally distributed, and *p* ≤ 0.05 indicates that the data were not normally distributed. In this case, all of the data used for cost analysis were not normally distributed. In order to compare the non-POM and the POM groups, the independent *t*-test was applied. *p* ≤ 0.05 was considered statistically significant. 

## 3. Results

### 3.1. Study Cohort

A total of 205 patients were admitted to the three wards throughout the study period who were all have been screened, but only 55 percent (n = 112) of these patients were on regular medications. After following up with these patients, only 104 patients were eligible for the study, out of which 8 patients were excluded because they were admitted longer than 21 days. From the pattern of prescribing, dispensing and follow-up with their medication administration chart via HIS, it was found that 63 (60.6%) of the 104 patients did not bring their own regular medications during admission, while the other 41 (39.4%) patients brought their own medications. The summary of baseline characteristics of the study sample is given in Table 1.

The mean length of stay for the non-POM group was not different from the POM group, with 7.3 ± 4.3 days and 6.4 ± 3.7 days, respectively. As shown in Table 2, the pattern of regular medications used during hospitalization was not significantly different for both groups.

### 3.2. Economic Analysis of Medication Used

The differences in cost spent per patient for each group are summarized in Table 3. The finding shows that the hospital spent approximately USD 21.60 per patient for the non-POM group, which sums up to a total of USD 1363.29 for the group. Whereas for the POM group, it was estimated that the hospital spent USD 28.40 per patient, which totaled to USD 532.70 for the respective group within the period of the study. Collectively, a significant cost had been saved from the use of POM during hospitalization, which was USD 625.04, and a total cost of USD 759.51 was possible to be saved if the use of POM was encouraged. The cost of medications used per patient for both groups was USD 295.89 and USD 443.75 for the non-POM group and POM group, respectively, per 100 patient days, in which the total costs were USD 18,674.90 and USD 18,074.08, respectively, for each group.

Table 4 shows the top 10 medications used in the wards during the study period for both the POM group and the non-POM group. Omeprazole was the most-used medication in the wards. The pattern of medications used for both groups was different, but both groups had furosemide and different types of heart medications as the top medications used in the wards.

Throughout the study period, approximately USD 157.31 value of ward stock medication was used per month, while the sum of the ward stock value kept in the medical and surgical ward was approximately USD 1554.2. On the other hand, ward stock with a cost of USD 18.28 was found to be expired within the duration of the study. Out of which, USD 16.65 was contributed by the medical ward. The wastage refers to the amount of medication that was unused and left in the trolley expired. The pattern of ward stock use in the wards is in Table 5.

## 4. Discussion

This is the first study that has investigated the cost impact of POM, unit-use and ward stock medication use during hospitalization. According to the previous study of POM use in Denmark’s surgical wards, there was no significant difference between the medication cost per patient for using POM with the traditional medication system, estimated to be USD 2.03 (95% CI: −0.57, 4.63, *p* = 0.131) [16]. Similar to this study, the supplied medication cost per patient was not significantly different as the mean difference was USD 8.60 (95% CI: −3.95, 27.47, *p* = 0.141). Although it was expected that the use of POM should reduce the cost spent by the hospital, as less medication would be supplied by the pharmacy as the patients brought their own medications, as in one of the preceding studies which the implementation of POM in Malaysia has shown a significant reduction in the total cost of USD 114.27 per patient per year [3]. There is a full ten-year record (2006–2016) of returned medicines from hospital wards and inpatient pharmacies from the main tertiary hospital in Brunei. Out of the total USD 2.7 million worth of returned medications, 53.8% were eventually reused; hence approximately USD 127k of medication is wasted per year [1,2]. As a matter of fact, the Ministry of Health of Brunei Darussalam’s annual expenditure has reached USD 176k for medical and pharmaceutical products, as stated in the recent database of 2020 [17]. Recently, many cost-saving alternatives and interventions have been introduced to reduce the problem of escalating the cost of wasted medications due to unused medication at home [9].

There are various potential reasons why the use of POM in the POM group did not show a significant difference in terms of the cost of medication supplied by the pharmacy in the non-POM group. Foremost, complex patients in the POM group, such as those who have multiple chronic diseases, may require the addition of new medication during admission. In addition, some patients might be stopped from taking some of their medications due to the reluctance of the physicians to increase the intensity of the long-term treatment [18]. Hence, this may result in starting a new regimen and stopping prior regular medications, hence increasing the quantity of medication supplied by the inpatient pharmacy for the POM group. Issues of medication wastage were also found in this study, where expired medications were found in the wards’ trolleys. There are a few potential reasons causing this, such as keeping the excess medication supplied to the patients, although it was not a part of their ward stock, and lack of awareness or lack of staff in managing the medication of the ward stock. Since patient treatment needs may change throughout the admission, the possibility of changing medications and dosages is high, and this might result in leftovers if patients’ prescriptions were delivered in advance. To provide a solution for the poor management of the leftover medications in the wards, the utilization of a unit-dose system to supply medication in inpatient settings is recommended [19]. This means the inpatient pharmacy would supply the medications on a daily basis instead of supplying medications a few days in advance. Recently, a one-year study was conducted in Portugal by utilizing a unit dose to cut down the leftovers of oral antibiotic dispensing practices [19]. In this particular study, the overall savings of 3939 pharmaceutical units, corresponding to EUR 1032.99, was reported, which could give up to EUR 434,085.85 in monetary savings for the whole of Portugal [19]. 

The finding of this study revealed that the use of POM helped to save 54.03% of the total cost spent by the hospital for the POM group within the period of the study, amounting to USD 625.04 in financial cost. Thus, it was estimated that the hospital could potentially save up to 55.7% (USD 759.21) of the total cost spent in the non-POM group if POM is used. Although statistically, the cost saved and the potential cost saved by using POM were not significantly different, comparing the total cost that can be saved if POM was used during admission can help to reveal that this intervention can still aid in reducing a relevant value spent by the hospital. On another note, although there was no statistical difference between the lengths of stay between the two groups, the mean length of stay for the non-POM group was approximately one day longer than the POM group. The POM group might be more easily managed and encounter fewer medication errors, resulting in earlier hospital discharge. Another study also suggested that the excess LOS was usually caused by adverse drug events [20]. The excess length of stay ranged from 1.2 to 4.6 days in US studies and 2.3 to 3.5 days in European studies [20]. Considering the fact that there is a potential reduction in total cost spent by the hospital for patients with POM, this intervention seemed to have the capacity to augment the hospital’s cost management and healthcare system too.

Promising benefits have been suggested by the use of POM during admission. This includes the opportunity for better patient counseling and implementing continuity of care to improve medication adherence, which is one of the most common problems. A previous study found that the use of POM in diabetic patients has a positive effect in improving glycemic control by 0.79% [3]. On another point of view, some studies stated that the use of POM might also reduce medication errors during admission since patients are more familiar with the treatment regimen and the generic brand used [14].

Often in the wards, patients were stopped from taking their regular medications prior to admission and started with a new regimen. However, starting or hastily adding a new treatment does not necessarily provide an advantage to patients. It was unequivocal that the addition of more medications increases the risk of having a drug–drug interaction [21]. For instance, in one of the studies, 40% of new medications prescribed during admission were discontinued when discharged, and medication that was changed for 10% of the patients was still within the same class [22]. Changing the patient’s treatment may also lead to the abandonment of the previous prescriptions, thus contributing to medication wastage, especially if not disposed of properly [9]. Another problem that may arise with the change in treatment might include medication error, as it was found that a minimum of one error was found in 12% of the warded patients with a newly changed prescription [7]. One of the potential errors was caused by the omission of crucial medications, especially in chronic patients [7]. This highlighted that unnecessary medication expenditure can lead to a cost burden for the healthcare system due to potential medication wastage [23]. Thus, recommending the use of POM during admission might help to prevent the aforementioned issues.

Nevertheless, the implementation of POM in inpatient settings is time and labor intensive in adapting to the changes in the workflow system, and it requires the involvement of the patients and other healthcare professionals. Although, in this study, the labor cost was not evaluated, which can contribute to a significant impact of using POM in inpatient settings. The use of POM also has the potential to cause medication errors as the quality of the medication brought by patients cannot be assured, and the packaging might be unrecognizable to nurses [14]. Therefore, continuous assessment is required in order to analyze the safety and feasibility of implementing this initiative in inpatient settings.

The implementation of the workflow system and standard operating procedures by involving a pharmacist in managing patients’ medications in the wards and to complete history taking and medicine reconciliation might help in ensuring effective patient care and reducing the prevalence of medication errors, such as dosing errors, omissions, duplications or drug interactions, during the hospitalization period. It has been proven in previous studies that the use of a patient’s own medication during hospitalization has been recognized for its value in completing medicine reconciliation, leading to a more precise drug profile [24]. A study in Texas, United States, found that the implementation of a workflow management system in a children’s hospital for eight months helped to reduce the error rate by 1.6% [25]. In another study, it was also mentioned that when a pharmacist takes charge of the physicians and nurses in managing the patient’s medications in the wards, the time spent by the healthcare workers on the medication process was reduced to 5.2 h per patient (from more than 112.7 h per patient); this translated to a cost saving of EUR 1219 per 100 patients [14]. In addition, the use of an automated dispensing system such as e-HIS might further help reduce medication error, time and cost by optimizing the inventory management and refill system [26]. Generally, the role of pharmacists is important in inpatient settings to assist in rationalizing medication prescribing and reducing drug–drug interactions, particularly when patients are on medications that are potentially inappropriate or unnecessary. Additionally, the involvement of a pharmacist in managing patients’ medications in the wards can help improve the healthcare system in delivering better patient care, hence reducing the time, cost and medication errors.

Medication errors should be avoided wherever possible as they can lengthen the period of admission, cost and may cause mortality [27]. Thus, it can be suggested that the effect of POM during admission can be further investigated in future research, such as the effects of using POM toward patients’ knowledge and understanding of their medication regimens. Furthermore, the results of the research study can be used as a reference source in order for healthcare authorities to implement the POM policy in the future. Before integrating this intervention as a practice in inpatient settings, corresponding interventional studies are required to assess and refine this process. Proper patient education might be adequate to instill awareness in using their own medications to optimize health expenditure.

### Strengths and Limitations and Implications for Practice

This study only focuses on the cost impact of POM use during hospitalization and not the influence of using POM exclusively. Hence only limited aspects were observed. In addition, any adverse drug events that occurred after discharge for both groups were not assessed in this study in which there might be several benefits that the researchers have missed from the use of POM in inpatient settings. Since the prevalence of post-discharge adverse events was 20% in discharged patients, it should be studied in both the non-POM and POM groups to see if the use of POM during hospitalization can reduce the prevalence of post-discharge adverse events [28]. Furthermore, the labor cost of physicians and nurses in managing the patients’ medications was not assessed in this study, therefore, contributing to the limitations of this study. Hence, the cost impact of POM use during admission and the true effects may be overestimated or underestimated. Concomitantly, the labor cost is important to assess to see the true impact of using POM on the healthcare system as a whole. This can be evaluated in a future study to improve the current study. Nevertheless, the researchers believed that the limitations were counterbalanced by the strengths of the study. In this pilot study, to test the feasibility of a prospective observational study, we were able to involve 104 patients over a study period of 8 weeks. Of note, three full-time researchers thoroughly screened the medication utilization of the samples with the help of both e-HIS and the physical ward medication administration chart enabled the researchers to closely examine the medications administered to the patients daily.

## 5. Conclusions

The utilization of POM during admission is capable of reducing the cost spent by the hospital by at least 50% if patients bring their own medications from home, albeit it is not statistically significant due to the small sample size. With the use of POM, the total time spent on the medication process can be reduced significantly for each patient, which, when translated to cost, will save a significant value. The findings in this study are paramount in determining the development of new guidelines or standard procedures in the subsequent management of medication in an adult ward setting.

## Figures and Tables

**Table 1:** Patient characteristics of study sample (n = 104).

Variable	n (%)	Mean (SD)
**Age (years) ^a^**	-	63.2 (15.8)
**Length of stay (days) ^a^**		6.9 (4.1)
**Gender ^b^**		
**Male**	62 (59.6)	
**Female**	42 (40.4)	
**Category ^b^**		
**Non-POM Group**	63 (60.6)	
**POM Group**	41 (39.4)	
**Number of regular medications per patient ^a^**		6.7 (3.4)

^a^ Mean (SD); ^b^ Categorical data, n (%).

**Table 2:** Characteristics of POM versus Non-POM Groups.

	POM Group	Non-POM Group	Significance (*p*-Value)
**Number of patients ^b^**	41 (39.4)	63 (60.6)	*p* ≥ 0.05 *
**Length of stay ^a^**	6.4 (3.7)	7.3 (4.3)	*p* ≥ 0.05 *
**Number of regular medications per patient ^a^**	7.2 (3.7)	6.4 (3.1)	*p* ≥ 0.05 *
**Number of regular medications used during admission per patient ^a^**	6.3 (3.3)	5.1 (3.2)	*p* ≥ 0.05 *
**Total number of inpatient medications used during ^a^ admission per patient ^a^**	8.68 (3.6)	8.27 (3.3)	*p* ≥ 0.05 *

* Independent *t*-test; ^a^ Mean (SD); ^b^ Categorical data, n (%).

**Table 3:** Mean ± SD economic analysis per patient.

Cost (USD)	Non-POM (n = 63)	POM (n = 41)	*p*-Value
**Medications prescribed**	21.6 (34.2) ^a^	28.4 (26.8)	*p* ≥ 0.05 *
**Medications supplied**	21.6 (34.2) ^a^	13.0 (18.3)	*p* ≥ 0.05 *
**Saving for using POM**	Not applicable	15.4 (20.1)	Not applicable
**Potential saving if POM were used**	12.1 (16.5)	Not applicable	Not applicable
**Cost saved/potential cost saved**	12.1 (16.5)	15.4 (20.1)	*p* ≥ 0.05 *

* Independent *t*-test; ^a^ same value because all prescribed medications were supplied due to no POM.

**Table 4:** Top 10 medications used in the wards for POM and non-POM groups.

No	POM Group	Quantity (n)	Non-POM Group	Quantity (n)
	Name of Medications		Name of Medications	
**1**	Omeprazole 20 mg	157	Omeprazole 20 mg	659
**2**	Metformin HCl XR 500 mg	88	Calcium Carbonate 1250 mg	284
**3**	Metformin HCl 500 mg	77	Paracetamol 500 mg	267
**4**	Doxazosin 4 mg	74	Furosemide 40 mg	262.5
**5**	Atorvastatin 20 mg	63	Calcium Polystyrene Powder	230
**6**	Clopidogrel 75 mg	60	Perindopril Erbumine 4 mg	224.5
**7**	Essential Phospholipids + Vit B1 B2 B6 B12 E + Nicotinamide Caps	58	Amlodipine 5 mg	176.5
**8**	Furosemide 40 mg	57	Ferrous Fumarate 200 mg	165
**9**	Gabapentin 300 mg capsule	57	Aspirin 100 mg	157
**10**	Gliclazide MR 30 mg	55	Warfarin Sodium 1 mg	148

**Table 5:** Calculated cost of ward stock medication per day.

Wards	Ward Stock Medications Used (USD)	Ward Stock Medications Maintained in Ward (USD)
Medical Ward	2.80	40.63
Male Surgical Ward	1.55	5.76
Female Surgical Ward	0.90	5.42
